# Coping with methodological dilemmas; about establishing the effectiveness of interventions in routine medical practice

**DOI:** 10.1186/1472-6963-6-160

**Published:** 2006-12-13

**Authors:** Yvonne JFM Jansen, Roland Bal, Marc Bruijnzeels, Marleen Foets, Rianne Frenken, Antoinette de Bont

**Affiliations:** 1 Institute of Health Policy and Management, Erasmus MC Rotterdam, P.O.Box 1738, 3000 DR Rotterdam, The Netherlands; 2 Stichting Lijn 1 Haaglanden, P.O.Box 138, 2270 ACVoorburg, The Netherlands

## Abstract

**Background:**

The aim of this paper is to show how researchers balance between scientific rigour and localisation in conducting pragmatic trial research. Our case is the Quattro Study, a pragmatic trial on the effectiveness of multidisciplinary patient care teams used in primary health care centres in deprived neighbourhoods of two major cities in the Netherlands for intensified secondary prevention of cardiovascular diseases.

**Methods:**

For this study an ethnographic design was used. We observed and interviewed the researchers and the practice nurses. All gathered research documents, transcribed observations and interviews were analysed thematically.

**Results:**

Conducting a pragmatic trial is a continuous balancing act between meeting methodological demands and implementing a complex intervention in routine primary health care. As an effect, the research design had to be adjusted pragmatically several times and the intervention that was meant to be tailor-made became a rather stringent procedure.

**Conclusion:**

A pragmatic trial research is a dynamic process that, in order to be able to assess the validity and reliability of any effects of interventions must also have a continuous process of methodological and practical reflection. Ethnographic analysis, as we show, is therefore of complementary value.

## Background

The question has been raised to what extent evidence from controlled clinical trials on prevention interventions is of value in the routine clinical practice of primary care [1-6]. Explanatory trials, the randomised controlled trials (RCTs) measuring the treatments' efficacy, meet the criteria for valid evaluation through randomisation, recruiting a sufficiently large number of subjects and using control situations [7-9] plus reliable measurements. Pragmatic trials, as opposed to explanatory RCTs, measure the effectiveness of treatments in routine clinical practice [8,10,11]. In pragmatic trials, the definition of the treatments is more or less standardised to correspond with daily clinical decision-making. Additionally, the heterogeneity of patients is reflected, fewer exclusion criteria are used, and blinding, randomisation and control situations may not always be used. As Hotopf argues, pragmatic trials are preferable when health care provision and services are to be evaluated, because their external validity to the extent of their usefulness in routine clinical practice is not compromised [12]. In the literature, pragmatic trials are considered to provide a realistic alternative to conventional RCTs [10,11,13,14].

The way that researchers actually deal with conducting pragmatic trials remains largely unexplored. In fact, there have been few publications examining the work that goes into producing evaluative outcomes and managing pragmatic trials or, for that matter, on RCTs (e.g.[15]). In this paper we show how researchers balance between scientific rigour and localisation in pragmatic trial research. Our case is the Quattro Study, a pragmatic trial on the effectiveness of multidisciplinary patient care teams used in primary health care centres in deprived neighbourhoods of two major cities in the Netherlands for intensified secondary prevention of cardiovascular diseases (CVD). To increase the chance of implementation of this project, a major condition was that the research interference with daily care routines was kept to a minimum. As the project adopted a 'tailor made approach', the GPs and the supportive staff were asked to develop their own procedures for the program, albeit within preset conditions, as only they could develop guidelines that would fit into their specific local situation. Our main question was: How do researchers cope with the methodological dilemmas of localising the execution of the trial in the participating primary health care centres?

## Methods

We used an ethnographic design. By means of participant observations [16-18], the first author observed the work of two researchers, a data manager, and four research assistants from April 2003 till December 2004. In this period, we also observed 20 research progress meetings. All meetings, observations and conversations were transcribed. Minutes of the meetings, research protocols, documents and questionnaires used for the Quattro Study were collected. From April 2003 until December 2004, the first author observed four out of seven practice nurses in their daily work, each for five workdays. Throughout each observation, it was possible to ask questions or to request clarification. Transcripts were made immediately after leaving the health care centres. Audiotaped semi-structured interviews were held with the researchers, project leader, data manager and three practice nurses up to January 2006. All interviews were transcribed immediately after the interviews and were sent back to the interviewees for member check.

After the observation period, all transcripts, minutes, and research documents were analysed more in-depth. We analysed all information manually and thematically, establishing overarching categories.

### Study setting

Aim of the Quattro-study was to examine the effectiveness and cost-effectiveness of a multidisciplinary collaboration between a practice nurse, a peer health educator, the GP, and assistant in providing intensified preventive care in general practices located in deprived neighbourhoods. The Quattro Study was a randomised controlled trial (RCT) carried out in three primary health care centres located in the deprived neighbourhoods of Rotterdam and The Hague. Patients in the intervention group obtained Quattro-care and three-monthly assessments of the risk profile. Patients from control group A received usual GP care and three-monthly risk assessments and the GP as well as the patient were informed about the results of these measurements. It was thought to be ethically and practically unacceptable to assess a risk profile and not to inform the patient and GP about the results. However, this approach of assessing risk and informing patients and GP interferes with daily practice and may bias the results. Therefore, a blinded control group B was needed to quantify the effect of the risk assessments. This group received usual GP care and was measured once at the end of the study.

The follow-up period for the intervention and control group A was 12 months and the intervention programme lasted 9 months. Participants in the study were patients at high risk of developing CVD; i.e. patients with a modifiable part of the absolute 10-year risk equal or greater than 5% contributed by smoking, hypertension or hypercholesterolemia.

The intervention consisted of the formation of a primary care team in the general practice composed of the GP, assistant, practice nurse and peer health educator (Quattro-care). The intervention protocol was based on GP guidelines for hypertension, hypercholesterolemia, diabetes mellitus, smoking and obesity, and described the procedures for the intervention team (GP (treatment task), practice nurse (risk assessment, coordination and informative task), assistant (logistic task) and peer health educator (ethnic specific health education)). Although the main lines of the protocol were fixed for participating general practices (e.g. 4 structured team meetings of the Quattro-care team and 4 individual education sessions), the protocol allowed adapting to tailor the intervention to the individual practice needs and organisation.

The effectiveness of the multidisciplinary collaboration was assessed by comparing patients from the intervention group with those from control group A after one year follow-up with regards to the reduction achieved in the absolute 10-year risk of developing CVD.

Control group B, aimed to quantify the effect of structured risk assessments performed in control group A, was compared with control group A.

The study ran from August 2000 until December 2005. Complementary qualitative research was considered necessary during the execution of the trial to evaluate feasibility, implementation and experiences with Quattro-care of health care professionals, patients and researchers. Ethical approval for the Quattro Study, which also incorporated the complementary qualitative research, was obtained from the Health Ethics Board of Erasmus University Medical Center in Rotterdam.

## Results

### Pragmatic decisions

Patient recruitment was a major concern in the Quattro Study. The researchers had to adapt the inclusion procedure to overcome a too homogeneous composition in, and a shortage of, eligible patients. The research team decided to change the age parameters from 18–70 years of age to 30–70 years of age in order to ensure a more representable proportion of the target population (document selection criteria addendum 2). Changing the age parameters in a study performed in deprived neighbourhoods would prevent a possible over-representation of indigenous male patients with a high absolute risk of CVD, with little or no elevated cardiovascular risk factors (document selection criteria addendum 2). Focusing on the absolute 10-year risk alone for both male and female patients would result in excluding a large number of eligible, relatively young, predominantly female patients [19]. The inclusion of sufficient numbers of women, ethnic groups and the prevention of a possible over-representation of white males became important issues next to the risk of CVD. The adjustment of inclusion criteria enabled a more accurate representation of the patient population of the participating health care centres in the intervention.

The participating GPs were able to decide whether patients had to be protected from the stress of participation because of a too complex medical history (severe co-morbidity). The GPs vetoed 641 patients (document final report). The reasons for rejection were 'the patient being under care of a specialist', 'the patient being correctly monitored' or 'other reason:...' (document patient risk profile form). This elimination of patients proved to be a disproportionate part of the eligible group of patients and adopting the GP vetoes would result in a too small target population needed for the study. Besides, the amount of vetoed patients was unevenly distributed among the participating GPs, making future comparison and extrapolation of found effects difficult. To enable the intervention to represent routine medical decision-making the research team chose to review the vetoed patients and readmitted 43 patients. Eventually, from the selected 2,263 eligible patients between 30–70 years old and with at least one CVD risk factor, the researchers were able to include 1,665 eligible patients into the study (document final report).

### Pragmatic approach and systematic design

For the researchers, blinding patients and preventing contamination between intervention and control group A patients were major concerns for establishing the effectiveness of the study. Blinding patients for the health care professional, however, proved to be problematic. The problems started to develop as soon as the patients arrived at the centres for their appointments. For the assistants at the reception desks it was unclear to which group a particular patient belonged and 'mistakes' in the allocation of patients were made. Control group A patients were either seen as intervention group patients when they were not, or were referred back to the researchers without receiving Quattro care (field notes research progress meeting 10-09-2003 and 23-03-2004).

The research team decided to provide the health care centres with a list of names of the patients included in intervention and control group A. This point received an additional remark in the minutes: "Methodologically not really correct, but a concession" (minutes research progress meeting 10-09-2003). As the researchers provided name lists to the health care centres, the researchers endangered the internal validity of the project as they informed the health care centres about which patients belonged to which research group. In the study design the possibility of contamination was already incorporated, as the centres were responsible for the internal organisation of the intervention and adjusting the intervention to the local circumstances (document research proposal). The trial was, therefore, not badly designed; the research team was merely forced to make it workable for the centres.

### From a pragmatic to a systematic intervention

For the researchers the assessment of the effectiveness of the multidisciplinary care teams was the core of the whole project. They planned the individual patient education sessions given by the practice nurse and/or migrant health educator. Multidisciplinary team meetings attended by all four professionals were to follow each other continuously within the centres (document manual for intervention 2000). This part of the project differed between the care practices, as multidisciplinary team meetings were either organised (but irregular and without all health care professionals attending), limited to only practice nurses and GPs, or informal deliberations. Because all health centres appropriated the intervention towards their own local circumstances, the set-up of the intervention differed for each centre.

The adjustments in the centres endangered the establishment of the ultimate effect of the project and were seen as "seriously inconvenient for the study. This way, the trial becomes impure." (field note research progress meeting 11-05-2004). As a result, the research team increased its interference in the intervention by having the multidisciplinary team meetings stringently implemented for establishing the effect of the structural collaborative care opposed to regular care on the reduction of CVD risk less ambiguously. " [...] if we make *too many *concessions the results will drift away from the original idea, meaning we cannot say anything about the whole project at the end" (conversation researcher 01-06-2004). The team eventually developed a Quattro guideline for the health care centres to work with and organised regular supportive intervention progress meetings for the practice nurses and peer health educators.

The project leader, however, constantly tried to prevent research interference within the health care centres from happening because, for him, these differences in practice were not a problem but important for gaining insight into what kind of organisational preconditions primary health care must meet if the implementation of prevention projects is to be successful (conversation project leader 10-09-2003). "To be able to say anything about the effects of such a prevention project in real life practice, the trial has to have as little contact with the actual intervention as possible" (conversation project leader 10-09-2003).

### From a pragmatic to a systematic follow-up

For establishing the effectiveness of the project, the researchers needed to be informed by the professionals about the data from the follow-up. The needed data were the physical measurements of patients, like BMI, blood pressure, total cholesterol, and fasting capillary glucose levels (or HbA1c). Moreover, they also needed data to measure the (costs of the) intervention, such as time spent by all professionals on the specific parts of this project, i.e. intake, patient consultations, and multidisciplinary meetings (document research proposal).

Getting the professionals to register and deliver the data, however, proved to be problematic (minutes research progress meeting 20-01-2004). As a result the researchers took measures concerning the data collection. First, the research team decided that the forms used by the health care professionals should contain fixed data, so that the researchers could use these forms too (minutes research progress meeting 20-01-2004). They also decided " [...] the research assistants would have to resolve the lacunas in the research data" (field note research progress meeting 03-02-2004). However, some lacunas in data could not be resolved. Data was either not recorded, and thus the research assistants had to retrieve these missing data from the health care centres, or patients had not gone for their lab measurements, resulting in data not being retrievable at all.

The follow-up of patients in this way increasingly became important as measuring point to establish the effectiveness of the trial, underscoring the systematic nature of the data collection. As the promise of tailoring the intervention resulted in the professionals appropriating the follow-up procedures, informing the researchers about the meantime follow-up results was seen to be an interference with the daily routines. The researchers, however, had to increase their efforts to collect the meantime follow-up results in order to be able to establish the effectiveness of the intervention systematically, as missing data in trials entails a validity problem for analysing the ultimate estimate of effect. The question to what extent the opportunity of tailoring the intervention and its execution to the local circumstances of the health care centres would result in appropriations in data collection was, however, not addressed.

## Discussion

Executing a pragmatic trial is a continuous balancing act for the researchers. Researchers constantly balance between meeting methodological demands to produce a scientifically rigorous effectiveness study and applying a pragmatic approach to making feasible and implementing a preventive intervention in primary health care. Both systematic and pragmatic approaches proved to be difficult to retain. By means of ethnographic analysis, we showed that the researchers conducting the Quattro Study had to adjust the research to enable the intervention's uptake in routine primary care. The researchers adapted the inclusion procedure to overcome the homogeneous composition of, and a shortage in, eligible patients and provided the health care professionals the name lists of included patients in order to restrict the provision of care to research groups. Moreover, the researchers had to increase their interference in the pragmatic execution of the intervention to have the trial performed more systematically. The researchers increased their interference in the organisation of the intervention by having the multidisciplinary team meetings implemented stringently and by having the data collected as systematically as possible. Our contention is that this balancing act is not a feature of this specific trial, nor that it points at methodological weaknesses, but a structural dilemma for pragmatic trials.

Pragmatic trials, we showed in this paper, pose substantial challenges to investigators. Such trials hold the premise of being both 'pragmatic' (enabling the uptake of treatments in daily care by limning to routine clinical decision-making and incorporating the heterogeneity of patients and health care professionals) and 'systematic' (establishing the effectiveness of treatments by means of a scientific method of experimental design and predefined outcome measures). The constant interaction between research and primary care leads to continuous adjustments in research and intervention. Reconciling the tensions between the two different intellectual traditions, as indicated by Campbell et al [20] and illuminated by Marks [21] make the pragmatic and the systematic parts of pragmatic trials influence each other in opposite directions.

As pragmatic trial research is a dynamic process in which the parts of research and (health care) practice will be redefined repeatedly, a continuous process of methodological and practical reflection is imperative. Otherwise, pragmatic trials may end up in being just a contradiction in terms. So, we do not suggest to solely focus on practical research strategies for improving study design, trial execution and generalisability of results as advocated by Campbell et al. [20] and Ward [22]. Neither do we suggest to focus the research efforts solely on the development of meaningful evidence about routine care, as is suggested by MacPherson [9]. Stead, we suggest using qualitative (ethnographic) analyses to evaluate the continuous interference of research and care in pragmatic trials, especially to be able to assess the validity and reliability of any effects of interventions (see e.g. [23,24]). Such analyses, as we experienced with the Quattro case, help the researchers in both finding and accommodating diversions between the pragmatic and systematic aspects of pragmatic trial research.

We therefore consider the information acquired by qualitative research important in both formative [23] and process evaluations [23] of pragmatic trial projects. As we were able to provide the researchers with ethnographic information concerning, for example, the differences in organising the multidisciplinary team meetings and the follow-up procedures among and within the health care centres, we provided an additional reflexive dimension for making adjustments in both research and practice. In addition, ethnographic process evaluations explicate the sequence of actions [25] performed in pragmatic trial projects. Qualitative research not only provides reflection on the inevitability of adjustments in pragmatic trials, it also provides reflection on the consequences of these adjustments.

Our ethnographic analysis itself of course also does have certain limitations. One is that our process evaluation on conducting a pragmatic trial may be biased due to the fact we only observed one pragmatic trial case. Because we did not observe other researchers conducting other pragmatic trials, we are aware that our accounts may not be generalisable in all respects. However, we do argue that balancing pragmatism and systematisation are structural to all pragmatic trials, although this may take different forms in other pragmatic trials. Secondly, our accounts may also be biased due to the fact that systematic ethnographic observations in the Quattro Study only started when the implementation was already taking place. All information about the project prior to those observations came out of the project's archive and on the basis of interviews and may thus be subjected to out-of-context interpretations. However, we have tried to triangulate all data as much as possible to overcome these biases.

## Conclusion

Pragmatic trials on complex interventions in primary health care pose substantial challenges to investigators. As we have shown, pragmatic trial research consists of constant interaction between research and health care practices; this leads to adjustments in research with respect to what part of the study should be systematically performed and executed to answer to scientific demands and what part of the project could have a pragmatic set-up in the health care centres. In the practice of pragmatic trial research, parts of research and health care practice will be redefined over and over again. Because pragmatic trial research is a dynamic process, in order to be able to assess the validity and reliability of any effects of interventions, it must also have a continuous process of methodological and practical reflection. Ethnographic analysis, as we showed, is therefore of complementary value.

## Competing interests

The author(s) declare that they have no competing interests.

## Authors' contributions

YJ had the original idea for this article. YJ and AB drew up the manuscript. Ethnographic data collection on the execution of the pragmatic trial and the performance of the intervention in the health care centres was done by YJ. RB, MB, MF and RF took part in reviewing the manuscript. All authors read and approved the final version of the article.

## Pre-publication history

The pre-publication history for this paper can be accessed here:


